# Intermittent Hypoxia Upregulates the *Renin* and *Cd38* mRNAs in Renin-Producing Cells via the Downregulation of miR-203

**DOI:** 10.3390/ijms221810127

**Published:** 2021-09-19

**Authors:** Yoshinori Takeda, Asako Itaya-Hironaka, Akiyo Yamauchi, Mai Makino, Sumiyo Sakuramoto-Tsuchida, Hiroyo Ota, Ryuji Kawaguchi, Shin Takasawa

**Affiliations:** 1 Department of Biochemistry, Nara Medical University, 840 Shijo-cho, Kashihara, Nara 634-8521, Japan; y-takeda@naramed-u.ac.jp (Y.T.); iasako@naramed-u.ac.jp (A.I.-H.); yamauchi@naramed-u.ac.jp (A.Y.); m.makino@naramed-u.ac.jp (M.M.); ssumiyo@naramed-u.ac.jp (S.S.-T.); hiroyon@naramed-u.ac.jp (H.O.); 2 Department of Obstetrics and Gynecology, Nara Medical University, 840 Shijo-cho, Kashihara, Nara 634-8522, Japan; kawaryu@naramed-u.ac.jp; 3 Respiratory Medicine, Nara Medical University, 840 Shijo-cho, Kashihara, Nara 634-8522, Japan

**Keywords:** CD38, cyclic ADP-ribose, intermittent hypoxia, juxtaglomerular cell, miR-203, renin-angiotensin system, sleep apnea syndrome

## Abstract

Sleep apnea syndrome is characterized by recurrent episodes of oxygen desaturation and reoxygenation (intermittent hypoxia [IH]), and it is a known risk factor for hypertension. The upregulation of the renin-angiotensin system has been reported in IH, and the correlation between renin and CD38 has been noted. We exposed human HEK293 and mouse As4.1 renal cells to experimental IH or normoxia for 24 h and then measured the mRNA levels using a real-time reverse transcription polymerase chain reaction. The mRNA levels of *Renin* (*Ren*) and *Cd38* were significantly increased by IH, indicating that they could be involved in the CD38-cyclic ADP-ribose signaling pathway. We next investigated the promotor activities of both genes, which were not increased by IH. Yet, a target mRNA search of the microRNA (miRNA) revealed both mRNAs to have a potential target sequence for miR-203. The miR-203 level of the IH-treated cells was significantly decreased when compared with the normoxia-treated cells. The IH-induced upregulation of the genes was abolished by the introduction of the miR-203 mimic, but not the miR-203 mimic NC negative control. These results indicate that IH stress downregulates the miR-203 in renin-producing cells, thereby resulting in increased mRNA levels of *Ren* and *Cd38*, which leads to hypertension.

## 1. Introduction

Sleep apnea syndrome (SAS) is a highly prevalent sleep disorder characterized by the repetitive partial or complete collapse of the pharynx during sleep. It induces apnea and hypopnea, which often result in decreased oxygen saturation. A growing body of evidence suggests that SAS acts through recurrent episodes of oxygen desaturation and reoxygenation (intermittent hypoxia (IH)) to cause hypertension [1]. The pathophysiology of hypertension in cases of SAS is complex and is dependent on various factors. Among those factors, the upregulation of the renin-angiotensin system (RAS) is of critical importance.

RAS plays an important role in the regulation of both the extracellular fluid volume and the blood pressure. Increasing activity on the part of the RAS contributes to hypertension in SAS patients [2]. Several reports indicate that the components of the RAS, such as renin (Ren), are increased in an environment characterized by IH [3]. Ren is an essential enzyme in relation to the regulation of the RAS. It is secreted by the renal juxtaglomerular (JG) cells that are located in the afferent arteriole of the glomerulus. Ren is regarded as a primary determinant of the activity of the RAS because it accelerates the RAS through converting angiotensinogen into angiotensin I. Although the upregulation of Ren by IH has been reported [4], its gene expression mechanism remains unclear.

In addition, several studies have suggested the regulation of Ren to be controlled by the CD38-cyclic ADP-ribose (cADPR)-mediated signaling pathway [5]. cADPR serves as a second messenger in terms of controlling the intracellular Ca^2+^ concentrations. It activates the Ca^2+^ release from the endoplasmic reticulum via the ryanodine receptor (RyR)s [6,7,8,9]. CD38 is a type II glycoprotein that contributes to the synthesis of cADPR [10,11,12]. Several studies have described how the CD38-cADPR-mediated signaling pathway is related to the pathogenesis of various diseases [11,12,13]. It has also been reported that the pathway affects renin production and release in a prototype of the JG cells, namely As4.1 [14]. We focused on CD38 as a contributor to the regulation of Ren expression in IH.

The present study sought to investigate the gene expression of both Ren and CD38, as well as their regulation mechanisms in response to IH stress in Ren-producing cells.

## 2. Results

### 2.1. Gene Expression Levels of Ren and Cd38 in Human and Mouse Renin-Producing Cells Were Increased by IH

**Figure 1:**
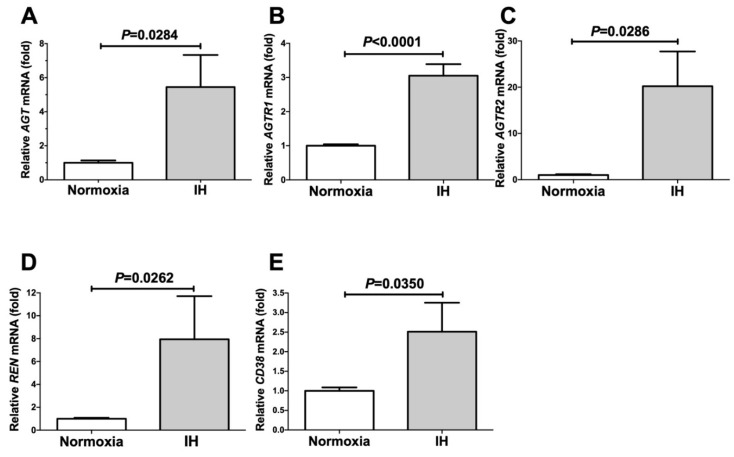
The mRNA levels of *AGT* (**A**), *AGTR1* (**B**), *AGTR2* (**C**), *REN* (**D**), and *CD38* (**E**) in the HEK293 cells subjected to normoxia or IH for 24 h. The levels of the RAS mRNAs were measured by means of a real-time RT-PCR using *β-actin* as an endogenous control. The data are expressed as the mean ± SE for each group of six independent experiments (n = 6). The statistical analyses were performed using a Student’s *t*-test.

We exposed human embryonic renal cell-derived HEK293 cells to normoxia or IH for 24 h. Following the IH treatment, we measured the mRNA levels of *angiotensinogen* (*AGT*), *angiotensin II receptor type 1* (*AGTR1*), *angiotensin II receptor type 2* (*AGTR2*), *renin* (*REN*), and *CD38* by means of a real-time reverse transcription polymerase chain reaction (RT-PCR). We found that the mRNA levels of *AGT*, *REN*, *AGTR1*, *AGTR2*, and *CD38* were all upregulated by IH (Figure 1). The mRNA levels of the components of both RAS and CD38, which have previously been reported to be related to renin expression [5,14,15], were significantly increased by IH.

**Figure 2:**
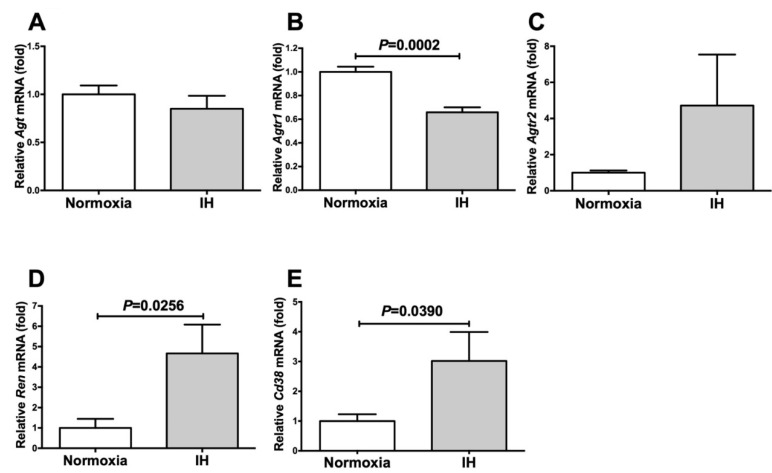
The mRNA levels of *Agt* (**A**), *Agtr1* (**B**), *Agtr2* (**C**), *Ren* (**D**), and *Cd38* (**E**) in the As4.1 JG cells subjected to normoxia or IH for 24 h. The levels of the mRNAs were measured by means of a real-time RT-PCR using *rat insulinoma gene* (*Rig*)*/ribosomal protein S15* (*Rps15*) as an endogenous control. The data are expressed as the mean ± SE for each group of six independent experiments (n = 6). The statistical analyses were performed using a Student’s *t*-test.

Next, we exposed mouse As4.1 JG cells to normoxia or IH for 24 h. Following the treatment, we measured the mRNA levels of *Agt*, *Agtr1*, *Agtr2*, *Ren*, and *Cd38* by means of a real-time RT-PCR. As shown in Figure 2, the mRNA levels of *Ren* and *Cd38* were significantly increased in the As4.1 JG cells in response to IH, although the *Agt*, *Agtr1*, and *Agtr2* levels were not.

**Figure 3:**
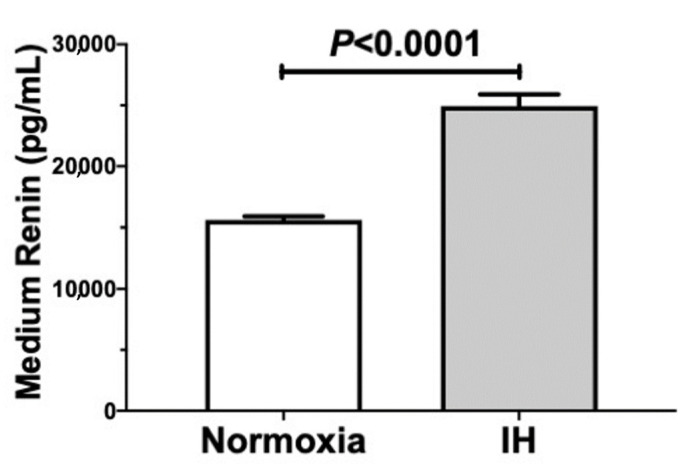
Concentrations of Ren in the As4.1 cell culture medium. The As4.1 cells were subjected to normoxia or IH conditions for 24 h. The medium Ren concentrations were measured by means of an ELISA. The data are expressed as the mean ± SE for each group of six independent experiments (n = 6). The statistical analyses were performed using a Student’s *t*-test.

**Figure 4:**
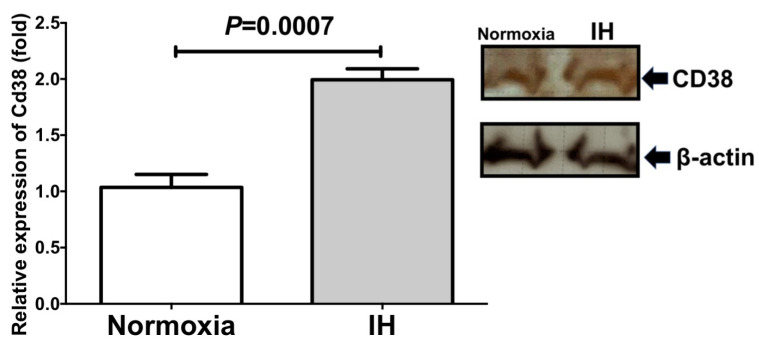
Relative protein expression of *Cd38* in the As4.1 JG cells subjected to IH. A representative immunoblot is shown in right panel. The relative expression of the *Cd38* is arbitrarily presented. The *Cd38* band densities were quantified using an image analysis and then normalized to β-actin, as measured in the same blot. Each bar represents the mean values of four independent experiments. The results are expressed as the mean ± SE in arbitrary units.

We further measured the level of Ren in the culture medium and the cellular *Cd38* level by means of an enzyme-linked immunosorbent assay (ELISA) and an immunoblot analysis, respectively. As shown in Figure 3 and Figure 4, IH significantly increased the medium Ren and cellular *Cd38* levels in the As4.1 cells.

### 2.2. Downregulation of Cd38 Attenuated the Ren Increase in As4.1 Cells Treated with Small Interfering RNA (siRNA) for Cd38

**Figure 5:**
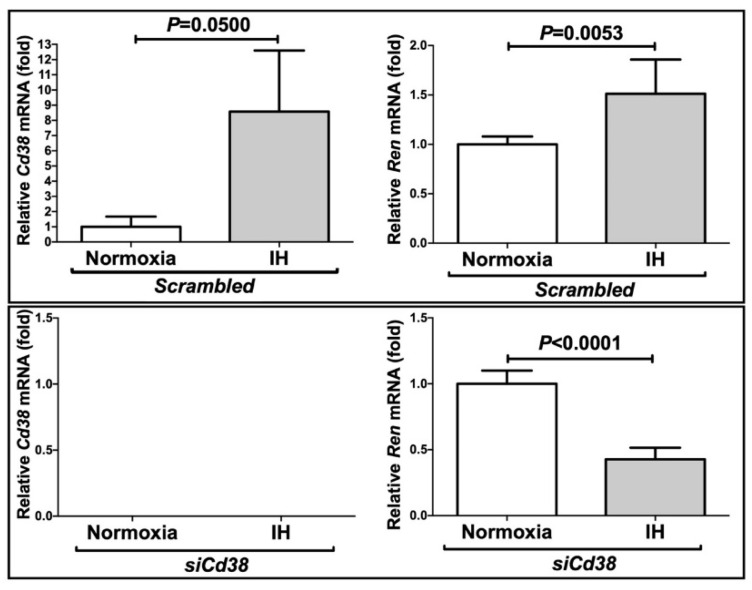
Effects of the siRNA against *Cd38* on the IH-induced gene expression of *Ren* and *Cd38*. The siRNA for *Cd38* was transfected into the As4.1 JG cells and the cells were then subjected to IH or normoxia for 24 h. The levels of the *Ren* and *Cd38* mRNAs were measured via a real-time RT-PCR using *Rig/RpS15* as an endogenous control. The data are expressed as the mean ± SE for each group of six independent experiments (n = 6). The statistical analyses were performed using a Student’s *t*-test. No *Cd38* mRNA was detected in the *siCd38*-introduced cells.

To investigate the mechanism of Ren expression in the As4.1 cells, the *Cd38* gene was knocked down by means of RNA interference. The expression levels of *Ren* and *Cd38* were significantly increased by IH in the presence of scrambled RNA. In contrast, the introduction of the siRNA for CD38 (*siCd38*) inhibited not only the IH-induced increases in the mRNAs for *Cd38,* but also the *Ren* levels in the As4.1 cells (Figure 5). These results indicated that the increases in the *Ren* levels observed in response to IH were caused by the *Cd38* expression levels. 

### 2.3. 8-Bromo-cADPR (8-Br-cADPR), a Cell-Permeable Antagonist of cADPR, Suppressed the Increases in the Ren and Cd38 Levels Induced by IH

**Figure 6:**
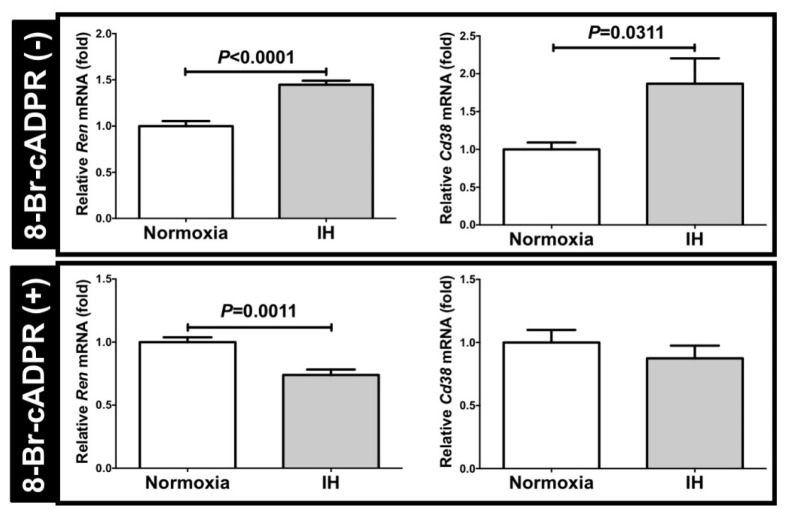
The mRNA levels of *Ren* and *Cd38* with or without the addition of the 8-Br-cADPR. There is no difference in *Ren* mRNA between 8-Br-cADPR (-) group and 8-Br-cADPR (+) group (1.062-fold increase in 8-Br-cADPR (+), *P* = 0.604). In contrast, the *Cd38* mRNA in 8-Br-cADPR (+) was increased 1.816-fold vs. 8-Br-cADPR (-) (*P* = 0.0024). Although the mRNA levels of *Ren* and *Cd38* were elevated in response to IH without the 8-Br-cADPR (8-Br-cADPR (-) controls), the elevation of the mRNAs disappeared following the addition of the 8-Br-cADPR (8-Br-cADPR (+)). The data are expressed as the mean ± SE for each group of six independent experiments (n = 6). The statistical analyses were performed using a Student’s *t*-test.

To confirm the correlation between the Ren expression and the CD38-cADPR-mediated signaling pathway, we added 8-Br-cADPR, the cADPR antagonist [9,16], into the As4.1 cell culture medium, and then subjected the cells to normoxia or IH for 24 h. Following the IH stimulation, we measured the mRNA levels of *Ren* and *Cd38* and determined that the increases in the *Ren* and *Cd38* mRNAs were suppressed (Figure 6). These results indicated that the increases observed in the *Ren* levels in response to IH were induced by the CD38-cADPR-mediated signaling pathway.

### 2.4. Gene Expression Levels of the Ryanodine Receptor (RyR)s Were Not Changed by IH

**Figure 7:**
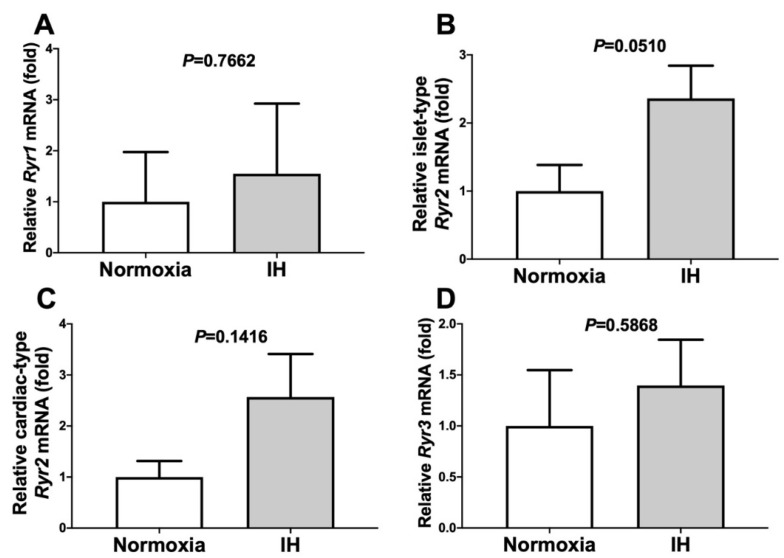
The mRNA levels of *Ryr1* (**A**), *islet-type Ryr2* (**B**), *cardiac-type Ryr2* (**C**), and *Ryr3* (**D**) in the As4.1 JG cells subjected to normoxia or IH for 24 h. In all cases, the mRNA levels were not elevated by IH (*P* = 0.7662, *P* = 0.0510, *P* = 0.1416, and *P* = 0.5868 in the *Ryr1*, *islet-type Ryr2*, *cardiac-type Ryr2*, and *Ryr3*, respectively). The data are expressed as the mean ± SE for each group of six independent experiments (n = 6). The statistical analyses were performed using a Student’s *t*-test.

The RyRs (i.e., RyR1, cardiac-type RyR2, islet-type RyR2, and RyR3) are intracellular Ca^2+^ release channels located on the endoplasmic reticulum. They represent important components of the CD38-cADPR-mediated signaling pathway [7,9]. To determine the involvement of the *Ryr*(s) in the elevation of *Ren* in response to IH, we measured the mRNA levels of the *Ryr*(s) following IH treatment. The mRNA levels of the *Ryr*(s) were not changed by IH (Figure 7). This result indicated that the elevation of *Ren* was not induced by changes in the *Ryr* expression level.

### 2.5. Promoter Activities of Ren and Cd38 Were Not Increased by IH

**Figure 8:**
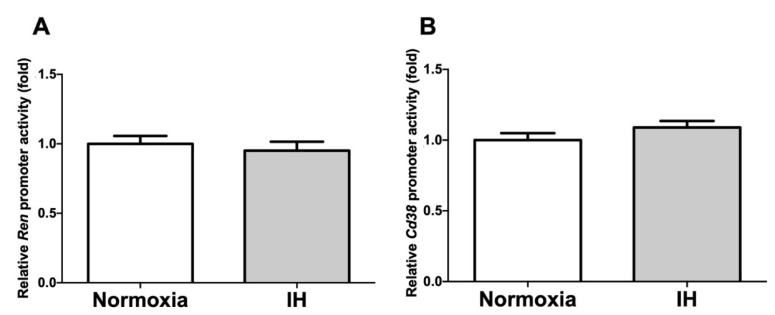
Promoter activities of *Ren* (**A**) and *Cd38* (**B**) in the As4.1 JG cells. Reporter plasmids prepared by inserting the promoter fragments of *Ren* (−4094 ~ +33) and *Cd38* (−4888 ~ +92) upstream of a firefly luciferase reporter gene in a pGL4.17 vector were transfected into the As4.1 cells. After the cells were exposed to either IH or normoxia for 24 h, they were lysed, and the promoter activities of *Ren* and *Cd38* were measured. All the data are represented as the mean ± SE of the samples of six independent experiments (n = 6). The statistical analyses were performed using a Student’s *t*-test.

To determine whether the IH-induced increases in the *Ren* and *Cd38* mRNA levels were caused by the activation of the transcription of the *Ren*/*Cd38* genes, a 4127 bp fragment containing 4094 bp of the mouse *Ren* promoter (−4094 ~ +33 of mouse *Ren* (L78789)) and a 4980 bp fragment containing 4888 bp of the mouse *Cd38* promoter (−4888 ~ +92 of mouse *Cd38* (NC_000071.6)) were fused to the luciferase gene of pGL4.17 and then transfected into the As4.1 JG cells. Following the IH stimulation, we measured the promoter activities of the *Ren* and *Cd38* and found that they were not changed (*p* = 0.5799 and *p* = 0.2114 in the *Ren* and *Cd38* promoters, respectively) by IH in the As4.1 cells (Figure 8). These results strongly suggested that the gene expression of *Ren* and *Cd38* in response to IH was not regulated by transcription.

### 2.6. The miR-203 Level Was Significantly Decreased by IH

**Figure 9:**
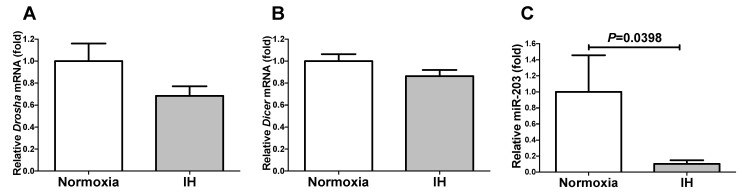
The levels of the *Drosha* mRNA (**A**), *Dicer* mRNA (**B**), and *miR-203* (**C**) of the As4.1 JG cells subjected to normoxia or IH for 24 h. The levels of the *Drosha* and *Dicer* mRNAs and the *miR-203* were measured by means of a real-time RT-PCR using *Rig/RpS15* (for the Drosha/Dicer) or *U6* (for the miR-203) as an endogenous control. The data are expressed as the mean ± SE for each group of six independent experiments (n = 6). The statistical analyses were performed using a Student’s *t*-test.

We considered it possible that the IH-induced upregulation of both *Ren* and *Cd38* was controlled post-transcriptionally. Therefore, we searched for targeted microRNA (miRNA) using the MicroRNA.org program (http://www.microrna.org/microrna/home.do; accessed on 16 May 2020), which revealed that the *Ren* and *Cd38* mRNAs have a potential target sequence for miR-203. There were no other miRNA candidates targeting both genes. We measured the miR-203 levels of the IH-treated cells by means of a RT-PCR and found that the levels were significantly lower than those of the normoxia-treated cells (*p* = 0.0398). There are a number of possible reasons why the miR-203 level was decreased by IH, including (i) the fact that the mRNA levels of some enzymes involved in miRNA biosynthesis/degradation are influenced by IH and (ii) the fact that the level of miR-203 is specifically decreased by IH, either via decreased biosynthesis or enhanced degradation. We measured the mRNA levels of both *ribonuclease type III* (*Drosha*) and *endoribonuclease Dicer* (*Dicer*), which are known to be involved in the biosynthesis of miRNAs [17,18], and found that their expression was unchanged by IH (Figure 9). These results suggested that miR-203 plays a key role in the post-transcriptional regulation of the mRNA levels of *Ren* and *Cd38*. To investigate whether the *Ren* and *Cd38* expression in response to IH is regulated by miR-203, the miR-203 mimic and non-specific control RNA (miR-203 mimic NC) were introduced into the As4.1 JG cells and then subjected to IH/normoxia exposure. The mRNA levels of *Ren* and *Cd38* were measured by means of a real-time RT-PCR.

**Figure 10:**
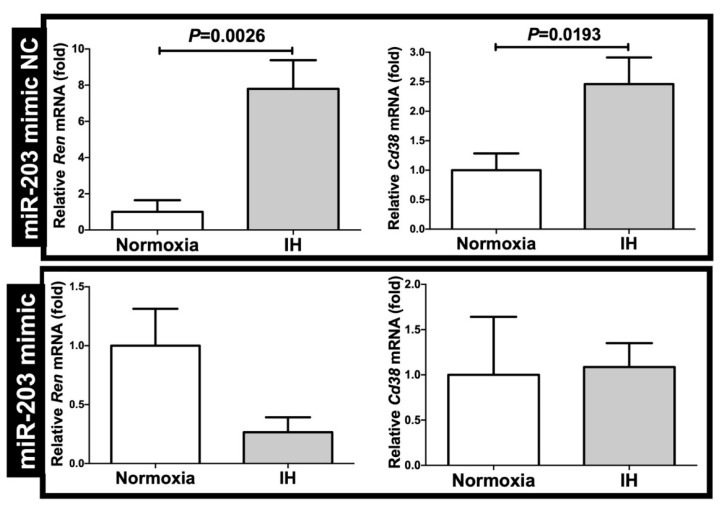
Effects of the miR-203 mimic transfection on the *Ren* and *Cd38* expression. The miR-203 mimic (5′-GUGAAAUGUUUAGGACCACUAG-3′, 5′-AGUGGUCCUAAACAUUUCACUU-3′) and the non-specific control RNA (miR-203 mimic NC) (5′-UUCUCCGAACGUGUCACGUtt-3′, 5′-ACGUGACACGUUCGGAGAAtt-3′) were synthesized by Nihon Gene Research Laboratories, Inc. (NGRL; Sendai, Japan). They were introduced into the As4.1 JG cells using Lipofectamine^®^ RNAiMAX just before the IH/normoxia exposure, and the mRNA levels of *Ren* and *Cd38* were measured by means of a real-time RT-PCR, as described in the Materials and Methods Section, using *Rig/RpS15* as an endogenous control. The data are expressed as the mean ± SE for each group of six independent experiments (n = 6). The statistical analyses were performed using a Student’s *t*-test.

As shown in Figure 10, we found that the IH-induced increases in the *Ren* and *Cd38* mRNAs were abolished by the introduction of the miR-203 mimic but not by the introduction of the miR-203 mimic NC. These findings indicated that IH stress downregulates the miR-203 level in mouse As4.1 JG cells, while the levels of the *Ren* and *Cd38* mRNAs are increased via the miR-203-mediated mechanism.

## 3. Discussion

SAS patients and their organs, tissues, and cells are exposed to IH but not to sustained hypoxia. Thus, we exposed JG cells, but not exposed JG cells, to IH by sustained hypoxia in this study and found that IH exposure induced increases in the *Ren* and *Cd38* mRNA levels in mouse JG cells. We further examined the mechanisms by which IH upregulates the mRNA levels of both *Ren* and *Cd38* and identified possible post-transcriptional miRNA-regulated mechanisms.

Recent epidemiological research has demonstrated that SAS may be associated with various metabolic dysfunctions, including dyslipidemia, cardiovascular diseases, insulin resistance, and hypertension. Additionally, in pregnant women, SAS may be a risk factor of gestational hypertension [19,20] and preeclampsia [21,22,23]. The pathophysiology of hypertension in relation to SAS is dependent on various factors, for example, the sympathetic tone, peripheral vasoconstriction, altered baroreceptor reflexes, and increased RAS activity [1]. In particular, there are numerous reports concerning RAS activity in SAS patients. More specifically, in SAS patients, the RAS activity has been found to be increased, which may cause blood pressure elevation [2,24]. Regarding renin expression in hypoxia, acute hypoxia stimulates renin secretion and renin gene expression in vivo [25,26], but chronic hypoxia suppresses renin gene expression [27]. Meanwhile, the expression of Ren in patients with SAS remains controversial. Some studies have reported that the Ren activity is not different between SAS patients and controls [28,29]. Conversely, several other studies have suggested that the Ren activity is higher in SAS patients than in controls [3]. In these studies, the severe SAS environment model could be associated with the higher Ren activity. According to our results, the mRNA levels of *Ren* were significantly higher in the IH condition (SAS model). This may be because our IH models imitated a more severer SAS environment than that considered in previous reports involving SAS patients.

We also focused on *Cd38* as a contributor to Ren expression. The mRNA levels of both *Cd38* and *Ren* were found to be increased in IH. *Cd38* is related to Angiotensin II activation and pathogenesis of cardiac hypertrophy and hepatic fibrosis [30,31]. Yi et al. reported that the expression of *Ren* was controlled by the CD38-cADPR-mediated signaling pathway in As4.1 cells [14]. To investigate the correlation between the expression of *Cd38* and *Ren*, we added the siRNA for *Cd38* to the As4.1 JG cells and found that the IH-induced *Ren* expression was significantly suppressed by the addition of *siCd38*. Moreover, we determined that the addition of 8-Br-cADPR suppressed the upregulation of both *Ren* and *Cd38* by IH. The mRNA levels of the *Ryr*(s) were unchanged by IH. These results indicated that the upregulation of *Ren* in the IH condition could be caused by the upregulation of *Cd38* (Figure 1, Figure 2, Figure 3 and Figure 4) and subsequent activation of the CD38-cADPR-mediated signaling pathway in As4.1 JG cells.

Additionally, we investigated the mechanisms by which IH upregulates the mRNA levels of *Ren* and *Cd38*. We found that the promoter activities of the genes were not increased by IH, which suggested that the IH-induced upregulation of the *Ren* and *Cd38* mRNAs is regulated during the post-transcriptional step. miRNAs are small non-coding RNAs (~22 nucleotides in length) that modulate gene expression by either translational suppression or the degradation of the mRNA through binding to the 3′-untranslated regions of the target genes in a base-pairing manner [32]. They affect the stability of their target mRNAs, resulting in changes in the amount of target mRNA, which is one of the mechanisms associated with post-transcriptional regulation. To date, a number of studies concerning the role of miR-203 have been performed in malignant neoplasms such as chronic myeloid leukemia [33], breast cancer [34], cervical cancer [35] and renal cell carcinoma [36], and IH-stimulated hepatocytes [37]. Several such studies have indicated that miRNAs are involved in the regulation of many biological processes (migration, metastasis, cell proliferation, apoptosis, chemosensitivity, etc.) in these various types of cells.

A few studies have addressed the correlation between miRNAs and hypertension in patients with SAS. For example, three plasma miRNAs (miR-378a-3p, miR-100-5p, and miR-486-5p) have been found to predict the blood pressure responses to continuous positive airway pressure treatment in patients with resistant hypertension and SAS [38]. In case of hypoxic pulmonary hypertension, miR-203 has been found to inhibit fibroblast growth factor 2 (FGF2), thereby resulting in a reduction in hypoxic pulmonary hypertension [39]. However, these studies did not indicate the involvement of miR-203 in SAS patients’ hypertension. In the present study, the decline of the miR-203 with a common target sequence in the *Ren* and *Cd38* mRNAs could have contributed to the worsening hypertension in the IH condition induced by the upregulation of the *Ren* and *Cd38* mRNAs.

In this study, the gene expression of *Ren* and *Cd38* was increased via the downregulation of the miR-203 level in the IH-treated JG cells. It is suggested that, in SAS patients, the upregulation of *Ren* and *Cd38* may induce hypertension, while miR-203 could play a crucial role in the regulation of such gene expressions.

## 4. Materials and Methods

### 4.1. Cell Culture

The utilized mouse JG As4.1 cells were purchased from the American Type Culture Collection (Manassas, VA, USA). The As4.1 cells and human embryonic kidney-derived HEK293 cells were grown in Dulbecco’s Modified Eagle Medium (FUJIFILM Wako Pure Chemical Corporation, Osaka, Japan) containing 10% (*v/v*) fetal calf serum (FCS), 100 units/mL penicillin G (FUJIFILM Wako), and 100 µg/mL streptomycin (FUJIFILM Wako), as described in prior studies [9,40,41]. The cells were exposed to either normoxia (21% O_2_, 5% CO_2_, and balanced N_2_) or IH (70 cycles of 5 min sustained hypoxia (1% O_2_, 5% CO_2_, and balanced N_2_) and 10 min normoxia) in a custom-designed, computer-controlled incubation chamber attached to an external O_2_-CO_2_-N_2_ computer-driven controller (O_2_ programmable control, 9200EX, Wakenyaku Co., Ltd., Kyoto, Japan), as described in previous works [37,42,43,44,45,46]. We used this in vitro model of IH, which resulted in fluctuations in the pressure of oxygen similar to the IH condition observed in patients with severe SAS, to repeatedly exposed the cells to severe hypoxemia followed by mild hypoxemia or normoxia (i.e., IH) [47]. We have previously reported that the magnitude of the IH expressed by SpO_2_ fluctuated between 75% and 98% and 50% and 80% in patients with SAS [42,45,46], which was nearly equivalent to the medium condition in the present study.

### 4.2. RT-PCR

The total RNA was isolated from the As4.1 and HEK293 cells using a RNeasy Plus Cell Mini Kit (Qiagen, Hilden, Germany), while the cDNA was synthesized from total RNA as a template using a High-Capacity cDNA Reverse Transcription Kit (Applied Biosystems, Foster City, CA, USA) as described in prior studies [37,43,48,49,50,51,52,53,54,55,56,57,58,59]. The real-time RT-PCR was performed using an SYBR^®^ Fast qPCR Kit (KAPA Biosystems, Boston, MA, USA) and a Thermal Cycler Dice Real-Time System (Takara Bio, Kusatsu, Japan). All the PCR primers were synthesized by Nihon Gene Research Laboratories, Inc. (NGRL; Sendai, Japan). The primer sequences for each primer set are described in Table 1 and Table 2. The RT-PCR was performed with an initial step of 3 min at 95 °C, followed by 45 cycles of 3 s at 95 °C, and then 20 s at 60 °C for the *AGT*, *REN*, *ACE*, *AGTR1*, *AGTR2*, *CD38*, and *β-actin*, as well as for the mouse *Agt*, *Ren*, *Ace*, *Agtr1*, *Agtr2*, *Cd38*, *Ryr1*, *islet-type Ryr2*, *cardiac-type Ryr2*, *Ryr3*, *Rig/Rps15*, *Dicer*, *Drosha*, *miR-203*, and *U6*. The RNA expression levels were normalized according to the mRNA level of the *β-actin* in human mRNAs and the level of *Rig/RpS15* in mouse mRNAs, while the *miR-203* level was normalized according to the *U6* RNA level.

### 4.3. Measurement of Ren in the Culture Medium by ELISA

The cells were subjected to either normoxia or IH for 24 h. Then, the culture medium was collected, and the Ren concentration was measured by using a Renin 1 (REN1) Mouse ELISA Kit (Thermo Fisher Scientific, Waltham, MA, USA) according to the supplier’s instructions.

### 4.4. Immunoblot Analysis

The immunoblot analysis was performed using an As4.1 cell extract (5 × 10^5^ cells), as described in previous studies [10,52,60], using an anti-Cd38 polyclonal antibody (Santa Cruz Biotechnology, Santa Cruz, CA, USA) raised against a peptide fragment of mouse Cd38 (residues 279–301 in [61]), an anti-β-actin monoclonal antibody (Sigma, St. Louis, MO, USA) raised against Ac-Asp-Asp-Asp-Ile-Ala-Ala-Leu-Val-Ile-Asp-Asn-Gly-Ser-Gly-Lys, and a SNAP id^®^ 2.0 Protein Detection System (Merck Millipore, Burlington, MA, USA). The band intensities were analyzed using ImageJ software (National Institute of Health, Bethesda, MD, USA), as previously described [52,62,63].

### 4.5. RNA Interference

The siRNA directed against the mouse *Cd38* was synthesized by NGRL. The sense sequence of the siRNA for the mouse *Cd38* was 5′-GGGCUACAUUGCUGAUGAUtt-3′ (corresponding to the 529–547 of NM_007646). The Silencer^®^ Select scrambled siRNA was purchased from Ambion and was used as a control. The transfection of the siRNA into the As4.1 cells was performed using the Lipofectamine^®^ RNAiMAX Transfection Reagent (Thermo Fisher Scientific, Waltham, MA, USA). The cells were each transfected with 5 pmol each siRNA in a 24-well culture dish, as described in prior works [37,43,48,49,53,54,55,56,57,58].

### 4.6. Addition of 8-Br-cADPR

The As4.1 cells were adjusted at 2 × 10^5^ cells/mL, and the 0.5 mL cell suspension was seeded into each well (24-well plate). After they were incubated at 37 °C overnight, the medium was replaced with fresh medium either containing 8-Br-cADPR (Sigma, St. Louis, MO, USA; finally to 100 µM) or without 8-Br-cADPR for each well. The cells were then further incubated at 37 °C in an IH/normoxia condition for 24 h. The cellular RNA preparation and real-time RT-PCR were performed as described in Section 4.2.

### 4.7. Construction of Reporter Plasmids and Luciferase Assay

The reporter plasmids were prepared by inserting the promoter fragments of the mouse *Ren* (−4094 ~ +33) and *Cd38* (−4888 ~ +92) upstream of a firefly luciferase reporter gene in the pGL4.17 vector (Promega, Madison, WI, USA). The reporter plasmids were then transfected into mouse the As4.1 cells using Lipofectamine^®^ 3000 (Invitrogen, Waltham, MA, USA), as previously described [54,55,56]. The cells were then exposed to either 70 cycles/24 h of IH (mimicking the As4.1 JG cells of SAS patients) or normoxia for 24 h. After the cells were exposed to IH, they were harvested and the cell extracts were prepared in an extraction buffer (0.1 M potassium phosphate, pH 7.8/0.2% Triton X-100; Life Technologies). To monitor the transfection efficiency, the pCMV•SPORT-βgal plasmid (Life Technologies, Carlsbad, CA, USA) was co-transfected in all the experiments at a 1:10 dilution. The luciferase activity was measured using a Pica Gene luciferase assay system (Toyo-ink, Tokyo, Japan) and was normalized according to the β-galactosidase activity, as described in earlier works [37,40,41,43,50,54,55,56,57,58,64].

### 4.8. MiR-203 Mimic Transfection

The miR-203 mimic (5′-GUGAAAUGUUUAGGACCACUAG-3′, 5′-AGUGGUCCUAAACAUUUCACUU-3′) and non-specific control RNA (miR-203 mimic NC) (5′-UUCUCCGAACGUGUCACGUtt-3′, 5′-ACGUGACACGUUCGGAGAAtt-3′) were synthesized by NGRL and then introduced into the As4.1 cells using Lipofectamine^®^ RNAiMAX (Thermo Fisher Scientific) [37,48,49,50,51,58,59] just prior to the IH/normoxia exposure. The mRNA levels of the *Ren* and *Cd38* were measured by means of a real-time RT-PCR, as previously described [37,43,44,48,50,53,54,55,56,57,58].

### 4.9. Data Analysis

The results are expressed as the mean ± SE. Statistical significance was determined by means of a Student’s *t*-test using GraphPad Prism software (GraphPad Software, La Jolla, CA, USA).

## Figures and Tables

**Table 1:** PCR primers (human) for the real-time RT-PCR.

Target mRNA	Primer Sequence
*REN* (NM_000537)	5′-AAATGAAGGGGGTGTCTGTGG-3′
	5′-AAGCCAATGCGGTTGTTACGC-3′
*CD38* (NM_001775)	5′-ACAAACCCTGCTGCCGGCTCTC-3′
	5′-GCATCGCGCCAGGACGGTCT-3′
*AGT* (NM_001382817)	5′-AACTGGTGCTGCAAGGATCT-3′
	5′-TCTCTCTCATCCGCTTCAAG-3′
*AGTR1* (NM_000685)	5′-ATCCACCAAGAAGCCTGCAC-3′
	5′-TGAAGTGCTGCAGAGGAATG-3′
*AGTR2* (NM_000686)	5′-CCTCGCTGTGGCTGATTTACTCCTT-3′
	5′-TTGCACATCACAGGTCCAA-3′
*β-actin* (NM_001101)	5′-GCGAGAAGATGACCCAGA-3′
	5′-CAGAGGCGTACAGGGATA-3′

**Table 2:** PCR primers (mouse) for the real-time RT-PCR.

Target mRNA/miR	Primer Sequence
*Ren* (NM_031192)	5′-CCTCTACCTTGCTTGTGGGATT-3′
	5′-CTGGCTGAGGAAACCTTTGACT-3′
*Cd38* (NM_007646)	5′-ACAGACCTGGCTGCCGCCTCTCTAG-3′
	5′-GGGGCGTAGTCTTCTCTTGTGATGT-3′
*Agt* (NM_007428)	5′-CACCCCTGCTACAGTCCATT-3′
	5′-GTCTGTACTGACCCCCTCCA-3′
*Agtr1* (NM_177322)	5′-GGCTGGCATTTTGTCTGGATA-3′
	5′-CTTTTCTGGGTTGAGTTGGTCT-3′
*Agtr2* (NM_007429)	5′-AGCTTACTTCAGCCTGCATT-3′
	5′-CAGCAACTCCAAATTCTTACACC-3′
*Ryr1* (NM_009109)	5′-AAGTCCCACAACTTTAAGCG-3′
	5′-TCTTCTTGGTGCGTTCCTG-3′
*Islet-type Ryr2* (NM_023868)	5′-GACAGTCGAGCGTGTCCTGGGTATA-3′
	5′-TGCTTAGAGAGTAGTTTGTGCCACA-3′
*Cardiac-type Ryr2* (NM_023868)	5′-GACAGTCGAGCGTGTCCTGGGTATA-3′
	5′-TGCTTAGAGAGTAGTTTGTGCCACA-3′
*Ryr3* (NM_001319156)	5′-AGAAGAGGCCAAAGCAGAGG-3′
	5′-GGAGGCCAACGGTCAGA-3′
*Rig/RpS15* (NM_009091)	5′-ACGGCAAGACCTTCAACCAG-3′
	5′-ATGGAGAACTCGCCCAGGTAG-3′
*Dicer* (NM_148948)	5′-ATGCAAAAAGGACCGTGTTC-3′
	5′-CAAGGCGACATAGCAAGTCA-3′
*Drosha* (NM_001130149)	5′-CTCTTTCCCACCCAGTGCTA-3′
	5′-TGGTCGTCGTAGTGCTTGAG-3′
*miR-203* (NR_029590)	5′-TCCAGTGGTTCTTGACAGTTCA-3′
	5′-GGTCTAGTGGTCCTAAACATTTC-3′
*U6* (XR_003953458)	5´-CGCTTCGGCAGCACATATAC-3′
	5′-AAATATGGAACGCTTCACGA-3′

## Abbreviations

AGT	Angiotensinogen
AGTR1	Angiotensin II receptor type 1
AGTR2	Angiotensin II receptor type 2
cADPR	Cyclic ADP-ribose
DICER	Endoribonuclease Dicer
DROSHA	Ribonuclease type III
ELISA	Enzyme-linked immunosorbent assay
FCS	Fetal calf serum
FGF2	Fibroblast growth factor 2
IH	Intermittent hypoxia
JG	Juxtaglomerular
miRNA	MicroRNA
RAS	Renin-angiotensin system
REN	Renin
Rig	Rat insulinoma gene
RpS15	Ribosomal protein S15
RyR	Ryanodine receptor
SAS	Sleep apnea syndrome
siRNA	Small interfering RNA

## References

[1] Ahmad M., Makati D., Akbar S. (2017). Review of and updates on hypertension in obstructive sleep apnea. Int. J. Hypertens..

[2] Jin Z.-N., Wei Y.-X. (2016). Meta-analysis of effects of obstructive sleep apnea on the renin-angiotensin-aldosterone system. J. Geriatr. Cardiol..

[3] Fletcher E.C., Bao G., Li R. (1999). Renin activity and blood pressure in response to chronic episodic hypoxia. Hypertension.

[4] Iturriaga R., Castillo-Galán S. (2019). Potential contribution of carotid body-induced sympathetic and renin-angiotensin system overflow to pulmonary hypertension in intermittent hypoxia. Curr. Hypertens. Rep..

[5] Xiong J., Xia M., Yi F., Abais J.M., Li N., Boini K.M., Li P.-L. (2013). Regulation of renin release via cyclic ADP-ribose-mediated signaling: Evidence from mice lacking CD38 gene. Cell. Physiol. Biochem..

[6] Galione A., Lee H.C., Busa W.B. (1991). Ca^2+^-induced Ca^2+^ release in sea urchin egg homogenates: Modulation by cyclic ADP-ribose. Science.

[7] Takasawa S., Nata K., Yonekura H., Okamoto H. (1993). Cyclic ADP-ribose in insulin secretion from pancreatic β cells. Science.

[8] Takasawa S., Akiyama T., Nata K., Kuroki M., Tohgo A., Noguchi N., Kobayashi S., Kato I., Katada T., Okamoto H. (1998). Cyclic ADP-ribose and inositol 1,4,5-trisphosphate as alternate second messengers for intracellular Ca^2+^ mobilization in normal and diabetic β-cells. J. Biol. Chem..

[9] Takasawa S., Kuroki M., Nata K., Noguchi N., Ikeda T., Yamauchi A., Ota H., Itaya-Hironaka A., Sakuramoto-Tsuchida S., Takahashi I. (2010). A novel ryanodine receptor expressed in pancreatic islets by alternative splicing from *type 2 ryanodine receptor* gene. Biochem. Biophys. Res. Commun..

[10] Takasawa S., Tohgo A., Noguchi N., Koguma T., Nata K., Sugimoto T., Yonekura H., Okamoto H. (1993). Synthesis and hydrolysis of cyclic ADP-ribose by human leukocyte antigen CD38 and inhibition of the hydrolysis by ATP. J. Biol. Chem..

[11] Takasawa S., Okamoto H. (2002). Pancreatic β-cell death, regeneration and insulin secretion: Roles of poly(ADP-ribose) polymerase and cyclic ADP-ribose. Int. J. Exp. Diabetes Res..

[12] Okamoto H., Takasawa S. (2021). Okamoto model for necrosis and its expansions, CD38-cyclic ADP-ribose signal system for intracellular Ca^2+^ mobilization and Reg ([Regenerating gene] protein)-Reg receptor system for cell regeneration. Proc. Jpn. Acad. Ser. B Phys. Biol. Sci..

[13] Okamoto H., Takasawa S. (2002). Recent advances in the Okamoto model: The CD38-cyclic ADP-ribose signal system and the regenerating gene protein (Reg)-Reg receptor system in β-cells. Diabetes.

[14] Yi F., Zhang A.Y., Li N., Zhang F., Xia M., Li P.-L. (2007). Role of cyclic ADP-ribose-Ca^2+^ signaling in mediating renin production and release in As4.1 cells. Cell. Physiol. Biochem..

[15] Sparks M.A., Crowley S.D., Gurley S.B., Mirotsou M., Coffman T.M. (2014). Classical renin-angiotensin system in kidney physiology. Compr. Physiol..

[16] Wei W.-J., Sun H.-Y., Ting K.Y., Zhang L.-H., Lee H.-C., Li G.-R., Yue J. (2012). Inhibition of cardiomyocytes differentiation of mouse embryonic stem cells by CD38/cADPR/Ca^2+^ signaling pathway. J. Biol. Chem..

[17] Francia S., Michelini F., Saxena A., Tang D., de Hoon M., Anelli V., Mione M., Carninci P., d’Adda di Fagagna F. (2012). Site-specific DICER and DROSHA RNA products control the DNA-damage response. Nature.

[18] Zhang J., Zhang X.-H., Wang C.-X., Liu B., Fan X.-S., Wen J.-J., Shi Q.-L., Zhou X.-J. (2014). Dysregulation of microRNA biosynthesis enzyme Dicer plays an important role in gastric cancer progression. Int. J. Clin. Exp. Pathol..

[19] Pamidi S., Pinto L.M., Marc I., Benedetti A., Schwartzman K., Kimoff R.J. (2014). Maternal sleep-disordered breathing and adverse pregnancy outcomes: A systematic review and metaanalysis. Am. J. Obstet. Gynecol..

[20] Watanabe M., Shinohara H., Kodama H. (2020). Nocturnal oxygen desaturation in the late third trimester of uncomplicated pregnancy for prediction of late-onset gestational hypertension. J. Obstet. Gynaecol. Res..

[21] Xu T., Feng Y., Peng H., Guo D., Li T. (2014). Obstructive sleep apnea and the risk of perinatal outcomes: A meta-analysis of cohort studies. Sci. Rep..

[22] Facco F.L., Parker C.B., Reddy U.M., Silver R.M., Koch M.A., Louis J.M., Basner R.C., Chung J.H., Nhan-Chang C.-L., Pien G.W. (2017). Association between sleep-disordered breathing and hypertensive disorders of pregnancy and gestational diabetes mellitus. Obstet. Gynecol..

[23] Jaimchariyatam N., Na-rungsri K., Tungsanga S., Lertmaharit S., Lohsoonthorn V., Totienchai S. (2019). Obstructive sleep apnea as a risk factor for preeclamsia-eclampsia. Sleep Breath..

[24] Foster G.E., Hanly P.J., Ahmed S.B., Beaudin A.E., Pialoux V., Poulin M.J. (2010). Intermittent hypoxia increases arterial blood pressure in humans through a renin-angiotensin system-dependent mechanism. Hypertension.

[25] Ritthaler T., Schricker K., Kees F., Krämer B., Kurtz A. (1997). Acute hypoxia stimulates renin secretion and renin gene expression in vivo but not in vitro. Am. J. Physiol..

[26] Krämer B.K., Ritthaler T., Schweda F., Kees F., Schricker K., Holmer S.R., Kurtz A. (1998). Effects of hypoxia on renin secretion and renal renin gene expression. Kidney Int..

[27] Schweda F., Blumberg F.C., Schweda A., Kammerl M., Holmer S.R., Riegger G.A.J., Pfeifer M., Krämer B.K. (2000). Effects of chronic hypoxia on renal renin gene expression in rats. Nephrol. Dial. Transplant..

[28] Muxfeldt E.S., Margallo V.S., Guimarães G.M., Salles G.F. (2014). Prevalence and associated factors of obstructive sleep apnea in patients with resistant hypertension. Am. J. Hypertens..

[29] Svatikova A., Olson L.J., Wolk R., Phillips B.G., Adachi T., Schwartz G.L., Somers V.K. (2009). Obstructive sleep apnea and aldosterone. Sleep.

[30] Guan X.H., Hong X., Zhao N., Liu X.H., Xiao Y.F., Chen T.T., Deng L.B., Wang X.L., Wang J.B., Ji G.J. (2017). CD38 promotes angiotensin II-induced cardiac hypertrophy. J. Cell. Mol. Med..

[31] Kim S.Y., Cho B.H., Kim U.H. (2010). CD38-mediated Ca^2+^ signaling contributes to angiotensin II-induced activation of hepatic stellate cells: Attenuation of hepatic fibrosis by CD38 ablation. J. Biol. Chem..

[32] Esteller M. (2011). Non-coding RNAs in human disease. Nat. Rev. Genet..

[33] Chakraborty C., Sharma A.R., Patra B.C., Bhattacharya M., Sharma G., Lee S.-S. (2016). MicroRNAs mediated regulation of MAPK signaling pathways in chronic myeloid leukemia. Oncotarget.

[34] Muhammad N., Bhattacharya S., Steele R., Ray R.B. (2016). Anti-miR-203 suppresses ER-positive breast cancer growth and stemness by targeting SOCS3. Oncotarget.

[35] Hasanzadeh M., Movahedi M., Rejali M., Maleki F., Moetamani-Ahmadi M., Seifi S., Hosseini Z., Khazaei M., Amerizadeh F., Ferns G.A. (2019). The potential prognostic and therapeutic application of tissue and circulating microRNAs in cervical cancer. J. Cell. Physiol..

[36] Braga E.A., Fridman M.V., Loginov V.I., Dmitriev A.A., Morozov S.G. (2019). Molecular mechanisms in clear cell renal cell carcinoma: Role of miRNAs and hypermethylated miRNA genes in crucial oncogenic pathways and processes. Front. Genet..

[37] Uchiyama T., Ota H., Itaya-Hironaka A., Shobatake R., Yamauchi A., Sakuramoto-Tsuchida S., Makino M., Kimura H., Takeda M., Ohbayashi C. (2017). Up-regulation of *selenoprotein P* and *HIP/PAP* mRNAs in hepatocytes by intermittent hypoxia via down-regulation of miR-203. Biochem. Biophys. Rep..

[38] Sánchez-de-la-Torre M., Khalyfa A., Sánchez-de-la-Torre A., Martinez-Alonso M., Martinez-García M.Á., Barceló A., Lloberes P., Campos-Rodriguez F., Capote F., Diaz-de-Atauri M.J. (2015). Precision medicine in patients with resistant hypertension and obstructive sleep apnea: Blood pressure response to continuous positive airway pressure treatment. J. Am. Coll. Cardiol..

[39] Wang L.-N., Yu W.-C., Du C.-H., Tong L., Cheng Z.-Z. (2018). Hypoxia is involved in hypoxic pulmonary hypertension through inhibiting the activation of FGF2 by miR-203. Eur. Rev. Med. Pharmacol. Sci..

[40] Murakami-Kawaguchi S., Takasawa S., Onogawa T., Nata K., Itaya-Hironaka A., Sakuramoto-Tsuchida S., Yamauchi A., Ota H., Takeda M., Kato M. (2014). Expression of *Ins1* and *Ins2* genes in mouse fetal liver. Cell Tissue Res..

[41] Nakazawa T., Takasawa S., Noguchi N., Nata K., Tohgo A., Mori M., Nakagawara K., Akiyama T., Ikeda T., Yamauchi A. (2005). Genomic organization, chromosomal localization, and promoter of human gene for FK506-binding protein 12.6. Gene.

[42] Ota H., Takasawa S., Yamauchi M., Yoshikawa M., Tomoda K., Kimura H. (2015). Intermittent hypoxia in pancreatic beta cells. Pancreat. Disord. Ther..

[43] Shobatake R., Takasawa K., Ota H., Itaya-Hironaka A., Yamauchi A., Sakuramoto-Tsuchida S., Uchiyama T., Makino M., Sugie K., Takasawa S. (2018). Up-regulation of *POMC* and *CART* mRNAs by intermittent hypoxia via GATA transcription factors in human neuronal cells. Int. J. Biochem. Cell Biol..

[44] Ota H., Tamaki S., Itaya-Hironaka A., Yamauchi A., Sakuramoto-Tsuchida S., Morioka T., Takasawa S., Kimura H. (2012). Attenuation of glucose-induced insulin secretion by intermittent hypoxia via down-regulation of CD38. Life Sci..

[45] Ota H., Fujita Y., Yamauchi M., Muro S., Kimura H., Takasawa S. (2019). Relationship between intermittent hypoxia and Type 2 diabetes in sleep apnea syndrome. Int. J. Mol. Sci..

[46] Kimura H., Ota H., Kimura Y., Takasawa S. (2019). Effects of intermittent hypoxia on pulmonary vascular and systemic diseases. Int. J. Environ. Res. Public Health.

[47] Murphy A.M., Thomas A., Crinion S.J., Kent B.D., Tambuwala M.M., Fabre A., Pepin J.-L., Roche H.M., Arnaud C., Ryan S. (2017). Intermittent hypoxia in obstructive sleep apnoea mediates insulin resistance through adipose tissue inflammation. Eur. Respir. J..

[48] Ota H., Itaya-Hironaka A., Yamauchi A., Sakuramoto-Tsuchida S., Miyaoka T., Fujimura T., Tsujinaka H., Yoshimoto K., Nakagawara K., Tamaki S. (2013). Pancreatic β cell proliferation by intermittent hypoxia via up-regulation of *Reg* family genes and *HGF* gene. Life Sci..

[49] Kyotani Y., Itaya-Hironaka A., Yamauchi A., Sakuramoto-Tsuchida S., Makino M., Takasawa S., Yoshizumi M. (2018). Intermittent hypoxia-induced epiregulin expression by IL-6 production in human coronary artery smooth muscle cells. FEBS Open Bio.

[50] Shobatake R., Itaya-Hironaka A., Yamauchi A., Makino M., Sakuramoto-Tsuchida S., Uchiyama T., Ota H., Takahashi N., Ueno S., Sugie K. (2019). Intermittent hypoxia up-regulates gene expressions of *peptide YY* (*PYY*), *glucagon-like peptide-1* (*GLP-1*), and *neurotensin* (*NTS*) in enteroendocrine cells. Int. J. Mol. Sci..

[51] Uchiyama T., Itaya-Hironaka A., Yamauchi A., Makino M., Sakuramoto-Tsuchida S., Shobatake R., Ota H., Takeda M., Ohbayashi C., Takasawa S. (2019). Intermittent hypoxia up-regulates *CCL2*, *RETN*, and *TNFα* mRNAs in adipocytes via down-regulation of miR-452. Int. J. Mol. Sci..

[52] Yoshimoto K., Fujimoto T., Itaya-Hironaka A., Miyaoka T., Sakuramoto-Tsuchida S., Yamauchi A., Takeda M., Kasai T., Nakagawara K., Nonomura A. (2013). Involvement of autoimmunity to REG, a regeneration factor, in patients with primary Sjögren’s syndrome. Clin. Exp. Immunol..

[53] Yamauchi A., Itaya-Hironaka A., Sakuramoto-Tsuchida S., Takeda M., Yoshimoto K., Miyaoka T., Fujimura T., Tsujinaka H., Tsuchida C., Ota H. (2015). Synergistic activations of *REG Iα* and *REG Iβ* promoters by IL-6 and glucocorticoids through JAK/STAT pathway in human pancreatic β cells. J. Diabetes Res..

[54] Fujimura T., Fujimoto T., Itaya-Hironaka A., Miyaoka T., Yoshimoto K., Yamauchi A., Sakuramoto-Tsuchida S., Kondo S., Takeda M., Tsujinaka H. (2015). Interleukin-6/STAT pathway is responsible for the induction of gene expression of REG Iα, a new auto-antigen in Sjögren׳s syndrome patients, in salivary duct epithelial cells. Biochem. Biophys. Rep..

[55] Tsujinaka H., Itaya-Hironaka A., Yamauchi A., Sakuramoto-Tsuchida S., Ota H., Takeda M., Fujimura T., Takasawa S., Ogata N. (2015). Human retinal pigment epithelial cell proliferation by the combined stimulation of hydroquinone and advanced glycation end-products via up-regulation of *VEGF* gene. Biochem. Biophys. Rep..

[56] Tsuchida C., Sakuramoto-Tsuchida S., Takeda M., Itaya-Hironaka A., Yamauchi A., Misu M., Shobatake R., Uchiyama T., Makino M., Pujol-Autonell I. (2017). Expression of *REG* family genes in human inflammatory bowel diseases and its regulation. Biochem. Biophys. Rep..

[57] Tohma Y., Dohi Y., Shobatake R., Uchiyama T., Takeda M., Takasawa S., Tanaka Y., Ohgushi H. (2017). *Reg* gene expression in periosteum after fracture and its in vitro induction triggered by IL-6. Int. J. Mol. Sci..

[58] Takasawa S., Tsuchida C., Sakuramoto-Tsuchida S., Takeda M., Itaya-Hironaka A., Yamauchi A., Misu M., Shobatake R., Uchiyama T., Makino M. (2018). Expression of human *REG* family genes in inflammatory bowel disease and their molecular mechanism. Immunol. Res..

[59] Akasaka J., Naruse K., Sado T., Uchiyama T., Makino M., Yamauchi A., Ota H., Sakuramoto-Tsuchida S., Itaya-Hironaka A., Takasawa S. (2019). Involvement of receptor for advanced glycation endproducts in hypertensive disorders of pregnancy. Int. J. Mol. Sci..

[60] Kato I., Yamamoto Y., Fujimura M., Noguchi N., Takasawa S., Okamoto H. (1999). CD38 disruption impairs glucose-induced increases in cyclic ADP-ribose, [Ca^2+^]_i_, and insulin secretion. J. Biol. Chem..

[61] Harada N., Santos-Argumedo L., Chang R., Grimaldi J.C., Lund F.E., Brannan C.I., Copeland N.G., Jenkins N.A., Heath A.W., Parkhouse R.M. (1993). Expression cloning of a cDNA encoding a novel murine B cell activation marker. Homology to human CD38. J. Immunol..

[62] Ikehata F., Satoh J., Nata K., Tohgo A., Nakazawa T., Kato I., Kobayashi S., Akiyama T., Takasawa S., Toyota T. (1998). Autoantibodies against CD38 (ADP-ribosyl cyclase/cyclic ADP-ribose hydrolase) that impair glucose-induced insulin secretion in noninsulin-dependent diabetes patients. J. Clin. Investig..

[63] Shervani N.J., Takasawa S., Uchigata Y., Akiyama T., Nakagawa K., Noguchi N., Takada H., Takahashi I., Yamauchi A., Ikeda T. (2004). Autoantibodies to REG, a beta-cell regeneration factor, in diabetic patients. Eur. J. Clin. Investig..

[64] Nakagawa K., Takasawa S., Nata K., Yamauchi A., Itaya-Hironaka A., Ota H., Yoshimoto K., Sakuramoto-Tsuchida S., Miyaoka T., Takeda M. (2013). Prevention of Reg I-induced β-cell apoptosis by IL-6/dexamethasone through activation of *HGF* gene regulation. Biochim. Biophys. Acta.

